# Study on expression of plasma sCD138 in patients with hemorrhagic fever with renal syndrome

**DOI:** 10.1186/s12879-018-3005-0

**Published:** 2018-03-01

**Authors:** Jing Li, Hong Du, Xue-Fan Bai, Xiao-Yan Wang, Ying Zhang, Hong Jiang, Ping-Zhong Wang

**Affiliations:** Center of Infectious Diseases, Tangdu Hospital, Air-force Military Medical University, 569 Xinsi Rd, Baqiao District, Xi’an, Shaanxi 710038 China

**Keywords:** Hantavirus, Hemorrhagic fever with renal syndrome, Soluble CD138, Prognosis

## Abstract

**Background:**

Until now, there is non-specific treatment, and exploring early and novel biomarkers to determine the disease severity and prognosis of hemorrhagic fever with renal syndrome (HFRS) would be of importance for clinician to take systematic and timely intervention. This study observed the expression of plasma sCD138, a soluble component shedding from the glycocalyx (GCX) to the circulating blood, and evaluated its predictive value on disease severity and prognosis of HFRS.

**Methods:**

One hundred and seventy-six patients with HFRS who were treated at our center between January 2011 and December 2013 were randomly enrolled in this study. The patients were divided into a mild-type group, a moderate-type group, a severe-type group and a critical-type group according to the HFRS criteria for clinical classification. Thirty-five blood samples from healthy subjects were obtained as the controls. The concentrations of sCD138 were detected using enzyme linked immunosorbent assay (ELISA). The levels of prothrombin time (PT), activated partial thromboplastin time (APTT), fibrinogen (Fib), albumin (ALB), alanine aminotransferase (ALT), aspartate aminotransferase (AST), white blood cells (WBC), platelets (PLT), glucose (GLU), blood urea nitrogen (BUN) and serum creatinine (Scr) in the samples were routinely tested. The levels of sCD138 among the different types were compared; the correlation among sCD138 and the laboratory parameters mentioned above were analyzed. The predictive effectiveness for prognosis of sCD138 was evaluated using the receiver operating characteristic (ROC) curve analysis.

**Results:**

Except for the mild-type, the levels of sCD138 in the moderate-, severe- and critical-type patients during the acute stage were significantly higher than that of the convalescent stage and the control (P<0.05). With the aggravation of the disease, the levels of sCD138 during the acute stage had an increasing tendency, while demonstrated no significant difference among the moderate-, severe- and critical-type patients (*P*>0.05). sCD138 was negatively correlated with Fib, PLT and ALB, and was positively correlated with WBC and AST (P<0.05). sCD138 demonstrated predictive effectiveness for prognosis with the area under the curve (AUC) of 0.778 (*P*<0.001).

**Conclusion:**

Dynamic detection of plasma sCD138 might be benefit to evaluating the disease severity and prognosis of the patients with HFRS.

## Background

As a rodent-borne disease caused by Hantavirus, hemorrhagic fever with renal syndrome (HFRS) usually manifest fever, hypotension, hemorrhage and kidney injury [1, 2]. Like sepsis, HFRS has general pathophysiologic characteristics of systemic inflammatory response syndrome (SIRS), while it also has unique clinical progresses usually through febrile, hypotensive, oliguric, diuretic and convalescent phases [3, 4]. In some critical-type HFRS, the febrile, hypotensive and oliguric phases can overlap, resulting in refractory shock, acute respiratory distress syndrome (ARDS), acute kidney injury (AKI), encephalopathy, severe blood clotting dysfunction and multiple organ dysfunction syndrome (MODS) [5].

Until now, there is non-specific treatment, and early discovery, diagnosis, monitoring and supportive management are still the major treatment principle of the disease [6]. Considering the evaluative and predictive ability of the disease severity of HFRS on laboratory parameters routinely tested is still poor because of the complicated pathophysiology, exploring early and novel biomarkers to determine the disease severity and prognosis would be of importance for clinician to take systematic and timely intervention [7].

Glycocalyx (GCX) plays an important role on regulating vasopermeability, promoting platelet aggregation and coagulation disturbance as a major ingredient of endothelial surface layer (ESL) [8], and the degree of GCX component degradation can reflect the degree of neutrophil adhesion, endothelial dysfunction, tissue injury and severity of sepsis [9]. Glucoprotein and proteoglycans (PGs) are principal components of GCX, and PGs has a core of protein, which can connect with negative charge GAG side chain [10]. Syndecans family are major components of PGs, including the CD138, also called syndecan-1, is composed of a ectodomain, a single membrane-spanning domain and a short endochylema domain with phosphorylated site [11, 12]. Under physiopathologic conditions, such as sepsis, chronic inflammation, acute decompensated heart failure and ischemical reperfusion injury [13–16], syndecan-1 can be shed from the GCX to the circulating blood as soluble type, which can be considered as an potential marker on predicting prognosis and diagnosis [17, 18].

As far as we know, there was no research on exploring the expression and relationship between soluble components of PGs and disease severity and prognosis of HFRS. In this observational prospective study, we observed the expression of plasma sCD138 in patients with HFRS and explore its predictive capacity for disease severity and prognosis.

## Methods

### Study participants

One hundred and seventy-six patients with HFRS that were treated at our center between January 2011 and December 2013 were randomly enrolled in this study. The demographic characteristics of the patients were collected from medical records. Patients who had diabetes, autoimmune disease, kidney diseases, hematological disease, cardiovascular disease, viral hepatitis and other infectious diseases such as sepsis infected by bacteria and fungus, dengue hemorrhagic fever, malaria, et al., were excluded.

The diagnosis of HFRS was made based on the positive enzyme linked immunosorbent assay (ELISA) result for both specific IgM and IgG antibodies against Hantaan virus (HTNV), an sole serotype epidemic in the study region as far as we know, in serum of the acute phase. The assay was performed using IgG/IgM capture ELISA kits and was analyzed via a multifunctional autoanalyzer (BIORAD-680, United States).

According to the HFRS criteria of clinical classification [19], the severity of HFRS was classified into four types: (1) mild, defined as patients who had kidney injury without oliguria and hypotension; (2) moderate, defined as patients who had uremia, effusion (bulbar conjunctiva), hypotension, hemorrhage (skin and mucous membranes), and AKI with typical oliguria; (3) severe, defined as patients who had severe uremia, effusion (bulbar conjunctiva and either peritoneum or pleura), hemorrhage (skin and mucous membranes), hypotension and AKI with oliguria (urine output of 50–500 mL/day) for ≤5 days or anuria (urine output of < 100 mL/day) for ≤2 days; and (4) critical, defined as patients who usually had one or more of the following complications compared with the severe patients: refractory shock (≥2 days), visceral hemorrhage, heart failure, pulmonary edema, brain edema, severe secondary infection, and severe AKI with oliguria (urine output of 50–500 mL/day) for > 5 days or anuria (urine output of < 100 mL/day) for > 2 days. Considering the clinical conditions that a majority of the survival patients had been discharged before the convalescent phase and the degree of AKI that was still severe during the early stage of the diuretic phase, the acute stage was defined as the period that included the febrile, hypotensive, and oliguric phases and the early three days of the diuretic phase in this study, and the convalescent stage was defined as the diuretic and convalescent phase except the early three days of the diuretic phase. Furthermore, the patients received follow up until 28 days after discharge, and the prognosis (death) in this study was defined as patient death during hospitalization or within the 28 days following discharge.

### Blood samples and detection

Three hundred and thirteen venous blood samples were drawn randomly from the patients including 176 samples during the acute stage, and 137 samples during the convalescent stage. Thirty-five blood samples from healthy subjects were obtained as the controls. All of the samples were stored in ethylenediamine tetraacetic acid (EDTA) tubes and were centrifuged at 2500 rpm for 10 min at 4 °C within 2 h after drawing. The plasma supernatant was pipetted carefully and transferred to polypropylene tubes and then stored at − 80 °C prior to analysis.

sCD138 levels were measured with commercially available ELISA kits (Quantikine, XiTang, Inc., Shanghai, China) and were tested using a multifunctional autoanalyzer (BIORAD-680, United States) according to the manufacturer’s instructions. Each sample was detected in duplicate. According to the difference of the clinical typing, some sample of the patients were further taken for multiple dilutions and a reasonable detection result was finally selected.

Eleven laboratory parameters including prothrombin time (PT), activated partial thromboplastin time (APTT), fibrinogen (Fib), albumin (ALB), alanine aminotransferase (ALT), aspartate aminotransferase (AST), white blood cells (WBC), platelets (PLT), glucose (GLU), blood urea nitrogen (BUN) and serum creatinine (Scr) were routinely tested using autoanalyzers (Sysmex XT-4000i, Japan; Hitachi 7600–100, Japan; hemagglutination analyzers PUN-2048B, Sysmex, Japan; blood gas analyzer, GEM Premier 3500). All the laboratory parameters mentioned above and sCD138 were measured in the same time frame.

### Statistical analysis

Statistical analysis was performed using SPSS 17.0 software (SPSS Inc., Chicago, IL, USA). Tables were created using Excel 2003 (Microsoft), and figures were created using GraphPad Prism 5 (GraphPad Software, SanDiego CA). Continuous variables are presented as the mean ± SD and were analyzed by Kolmogorov-Smirnov’s test for normal distribution and by Levene’s test for the homogeneity of variance. The variables among the four types were compared by the SNK test for normally distributed variables. The non-normally distributed variables are presented as medians with interquartile ranges and were compared by the non-parametric Kruskal-Wallis H test. The Nemenyi Rank test was used to compare the differences among the four types. The frequencies and percentages are given for qualitative variables, and the differences among the four types were tested using the Pearson’s chi-square test. Spearman’s correlation coefficient was used to determine the relationship between sCD138 and the laboratory parameters as mentioned above. The predictor values of sCD138 for disease prognosis were tested using receiver operating characteristic (ROC) curves and quantified by calculating the area under the ROC curve (AUC) and the 95% confidence interval (CI). A two-tailed *P* < 0.05 was considered statistically significant.

### Ethics statement

The study was approved by the Institutional Review Board of Tangdu Hospital. Before inclusion, the patients were informed about the objectives of this study, and they or their direct relatives agreed and signed the informed consent form before inclusion.

## Results

### Clinical typing and demographic characteristics for patients with HFRS

Of the enrolled patients, including 40 females and 136 males, 32 cases were classified as mild, 50 cases were classified as moderate,43 cases were classified as severe and 51 cases were classified as critical according to the HFRS criteria of clinical classification. Eighteen critical individuals died during the acute stage with fatality rate of 10.23%. There was no significant difference in the sex or age distribution among the groups (*P*>0.05) (Table 1).

**Table 1:** Demographic characteristics for patients with HFRS

	Mild group (*n* = 32)	Moderate group (*n* = 50)	Severe group (*n* = 43)	Critical group (*n* = 51)	Control group (*n* = 35)
^a^ Female, n (%)	8 (25.0)	12(24.0)	8 (18.6)	12 (23.5)	11 (31.4)
^b^ Age, years	40.27 ± 13.72	41.42 ± 13.23	41.24 ± 12.61	48.58 ± 11.56	41.15 ± 12.24

^a^Pearson’s χ^2^test: χ^2^ = 1.757, *P* = 0.780

^b^ANOVA: F = 2.428, *P* = 0.072

### Levels of sCD138 in patients with HFRS

**Fig. 1:**
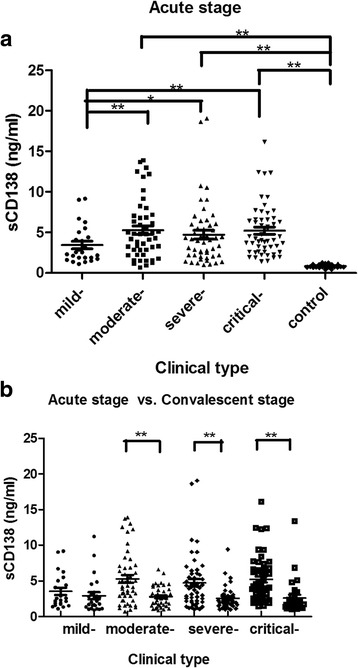
Levels of sCD138 during the clinical course in patients with HFRS. Except for the mild-type, the levels of sCD138 in the moderate-, severe- and critical-type patients during the acute stage were significantly higher than that of the convalescent stage (**b**.) and the control (**a**.), and with the aggravation of the disease, the levels of sCD138 had an increasing tendency, while demonstrated no significant difference among the moderate-, severe- and critical-type patients (**a**.). * *P*<0.05; ** *P*<0.001

The duration from disease onset to sample collection during the acute stage among the four groups was not significantly different (*P* = 0.312) (Table 2). Except for the mild-type, the levels of sCD138 in the moderate-, severe- and critical-type patients during the acute stage were significantly higher than that of the convalescent stage and the control (P<0.05), and with the aggravation of the disease, the levels of sCD138 during the acute stage had an increasing tendency, while demonstrated no significant difference among the moderate-, severe- and critical-type patients (*P*>0.05). The comparison of the sCD138 during the convalescent stage among the four types demonstrated no significant difference (P<0.05) (Table 2, Fig. 1).

**Table 2:** Levels of sCD138 and time frame of sample collection in patients with HFRS

	Mild group (*n* = 32)	Moderate group (*n* = 50)	Severe group (*n* = 43)	Critical group (*n* = 51)	Control group (*n* = 35)
sCD138, ng/mL					
^a^ Acute stage	3.45 ± 2.32	5.29 ± 3.64	4.73 ± 3.83	5.22 ± 3.19	0.819 (0.260)
^b^ Convalescent stage	2.71 ± 2.45*	2.78 ± 1.39*	2.54 ± 1.68*	2.56 ± 2.58*	0.819 (0.260)
Length of time (days)					
^c^ Acute stage	7 (4)	7 (3)	7 (3)	7 (4)	–
^d^ Convalescent stage	11.5 (4)	13 (3)	17 (10)	22 (10)	–

^a^Kruskal-Wallis H test: χ^2^ = 89.367, *P*<0.001. ^b^ Kruskal-Wallis H test: χ^2^ = 75.894, *P*<0.001

^c^Kruskal-Wallis H test: χ^2^ = 5.612, *P* = 0.312. ^d^ Kruskal-Wallis H test: χ^2^ = 45.344, *P*<0.001

*Comparison of the levels of sCD138 between the acute and convalescent stage: mild group, t = 0.873 *P* = 0.387; moderate group, t = 4.253 *P*<0.001; severe group, t = 3.253 *P* = 0.002; critical group, t = 3.748 *P*<0.001

### Spearman’s correlation analysis and ROC curves

**Fig. 2:**
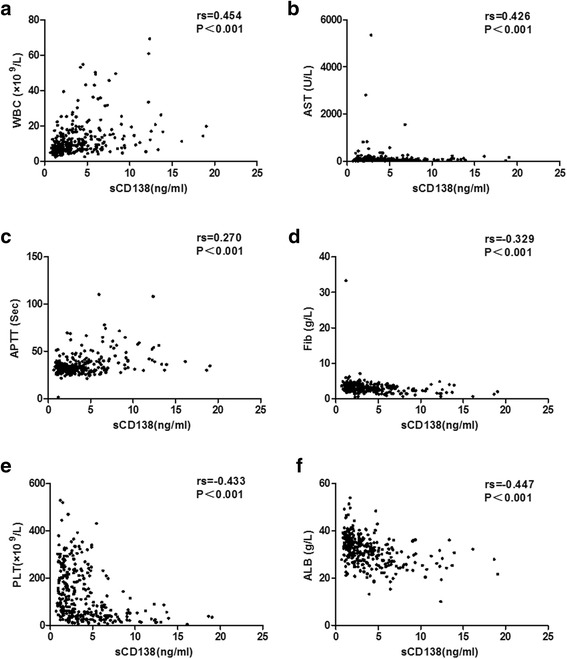
Correlation between sCD138 and WBC (**a**.), AST (**b**.), APTT (**c**.), Fib (**d**.), PLT (**e**.) and ALB (**f**.) in patients with HFRS by Spearman’s correlation analysis. sCD138 was obviously positively correlated with WBC and AST, and was negatively correlated with Fib, PLT and ALB (P<0.05). Abbreviations: sCD138, soluble CD138; WBC, white blood cells; PLT, platelets; ALB, albumin; AST, aspartate aminotransferase; APTT, activated partial thromboplastin time; Fib, fibrinogen

**Fig. 3:**
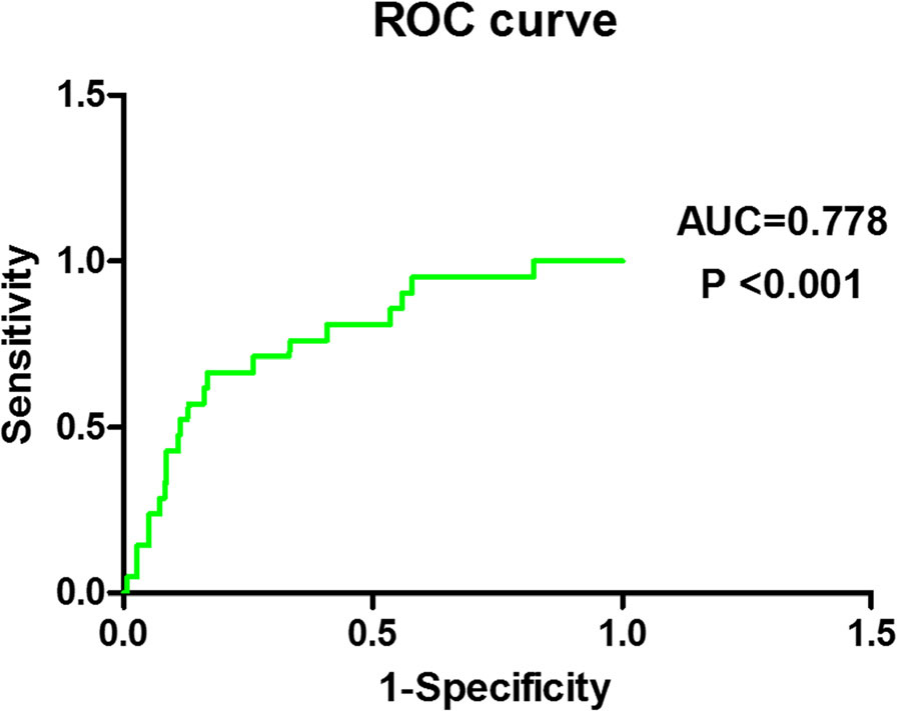
Predictive values for prognosis with sCD138 by ROC curve analysis. sCD138 showed predictive value of prognosis, with the AUC of 0.778 (P<0.001)

sCD138 was negatively correlated with Fib, PLT and ALB, and was positively correlated with WBC and AST, with correlation coefficients above 0.300 (P<0.05) (Table 3, Fig. 2). sCD138 demonstrated predictive value for prognosis with the AUC of 0.778 (*P*<0.001) (Table 4, Fig. 3).

**Table 3:** Spearman’s correlation analysis in patients with HFRS

Variables	sCD138	
	r	*P* value
WBC	0.454	<0.001
ALT	0.185	0.001
AST	0.426	<0.001
GLU	0.232	<0.001
PT	0.143	0.018
APTT	0.270	<0.001
Fib	−0.329	<0.001
PLT	−0.433	<0.001
ALB	−0.447	<0.001
BUN	0.235	<0.001
Scr	0.138	<0.001

Abbreviations: *r* correlation coefficient, *sCD138*, soluble CD138; *WBC*, white blood cells; *PLT,* platelets; *ALB*, albumin; *ALT*, alanine aminotransferase; *AST*, aspartate aminotransferase; *GLU*, glucose; *PT*, prothrombin time; *APTT,* activated partial thromboplastin time; *Fib*, fibrinogen; *BUN*, blood urea nitrogen; *Cr*, creatinine

**Table 4:** Predictive values for prognosis with sCD138 in patients with HFRS

Variables	AUC	^a^ *P* value	^b^ Cut off value	^c^ Sensitivity	^c^ Specificity	^c^ 95% Cl for AUC	
						Lower	Upper
sCD138	0.778	<0.001	5.868	66.7	83.2	0.678	0.877

Abbreviations: *AUC*, area under the curve; *CI*, confidence interval; *sCD138*, soluble CD138

^a^*P* value for calculated AUC in predicting death

^b^Units of the parameters were ng/ml

^c^Sensitivity, specificity and 95% CI are all presented as percentages

## Discussion

As an acute viral disease, HFRS has the basic clinical characteristics of sepsis [20], but it also has a unique pathophysiologic feature. The hypotensive phase of HFRS (e.g., low blood pressure and circulation collapse) usually occurs between day 3 and day 7 of the clinical course, and grave HFRS patients can manifest more severe leukemoid reaction, plasma leakage and coagulation disorders [21, 22] compared with septic patients, which would lead to massive bleeding, profound shock, severe tissue hypoperfusion and severe hypoxia, potentially rendering renal, cardiac, cerebellar and hepatic injury [23, 24]. Although there were some studies demonstrated that sCD138 can be considered as a novel marker on predicting prognosis in lung injury and sepsis patients [25–27], while as far as we know, until now, there was no research on exploring its expression and relationship with disease severity and prognosis of HFRS. Research of the expression of plasma sCD138 on different clinical types and courses would be benefit to illustrating its role on pathogenesis, disease severity and prognosis evaluation on HFRS by clinicians.

From this study, we observed that the levels of sCD138 in the moderate-, severe- and critical-type patients during the acute stage were significantly higher than that of the convalescent stage and the control, while the expression of sCD138 demonstrated no significant difference among the three types, which indicates that high levels of sCD138 could reflect the shedding of glycocalyx under systemic inflammatory response to a degree, which was similar with the patients with severe sepsis and septic shock [14, 28].

Our previous study [3, 4, 19] analyzed the levels of 12 routinely tested laboratory parameters in HFRS patients and demonstrated that WBC, PLT and ALB can be beneficial as early indicators of severity and prognosis in HFRS patients. High WBC can reflect the degree of inflammatory reaction; destruction or dysfunction of PLT (low PLT) [29] and low Fib can reflect the increasing risk of hemorrhage; while the low expression of ALB can represent the result of leakage from the damage vessel to the tissue interspace, which can reflect the loss of vascular integrity and the change of vascular permeability to a degree [24, 30]. In this study, we observed that sCD138 was obviously positively correlated with WBC, and was negatively correlated with Fib, PLT and ALB (P<0.05) (Table 3, Fig. 2), which not only provided supporting evidence on the major origin of sCD138 coming from the shedding of the PGs, but also illustrated that the destruction of GCX usually companied with more reactive inflammatory and immunity response, permeability change of vascular and clotting dysfunction.

In this study we also observed that plasma sCD138 were positively correlated with AST. Considering the high level of AST can manifest the abnormality of heart and liver function [4.19], which further indicated that the expression of plasma sCD138 can reflect the injury of multi-organ system and disease severity to a degree. Furthermore, in our study, the ROC analysis demonstrated that sCD138 showed predictive value of prognosis, with the AUC of 0.778 (*P*<0.001), which indicated that sCD138 had better predictive capacity for prognosis of HFRS and potential clinical application, like WBC, AST, PLT, ALB and HMGB-1 explored from our previous studies [2–4, 19].

As an observational prospective study, we got a meaningful conclusion that dynamic detection of plasma sCD138 might be benefit to evaluating the disease severity and prognosis of the HFRS patients, while there were still some limitations:

First, this study was conducted at a single center for infectious diseases. The length of time from collection of the blood samples was not unified or precise, considering the different clinical conditions and phases on admission in our center, and we can only define two periods: the acute and the convalescent stages. Although there was no significant difference in the median collection time of the samples in the acute stage, the dynamic change of the levels of plasma sCD138 can also be influenced by this variation and the pathologic inter-patient variability during the acute phase of infection.

Second, as we mentioned above, the relatively small number of cases and also the fragmentation of data collection made the statistical power small relatively. In this study, we were not able to collect more cases of HFRS to enlarge study samples in the last 3 years because of the lower incidence of HFRS in our region. Analyzing the major reasons, the interval from 2011 to 2013 was a period of city economy development, and the real estates construction led to destroyed ecological environment usually accompanied with the destruction of the rodents’ life cycle and environment, which cause a large numbers of rodents, especially the *Apodemus agrarius*, major source of infection, immigrate to the colonized region of the human and cause infection; furthermore, the negligence of HFRS vaccine injection in time on that interval was another reason. In the last 3 years, with the caution of local epidemiological investigation and active immunity of HFRS vaccine injection on population with inoculation rate above 85%, the HFRS incidence was obviously low. While, we should also be pay attention to the next large epidemic interval of HFRS in this region considering the descending valence of vaccine (usually sustain 3–5 years).

Furthermore, only 18 critical individuals died were enrolled in this study, the relative small number enrolled would influence the result of the ROC curve analysis. Finally, the clinical outcomes and classifications of the HFRS patients might be biased due to the lack of a more standardized protocol for the management of patients with HFRS until now.

## Conclusion

Dynamic detection of plasma sCD138 might be benefit to evaluating the disease severity and prognosis of the patients with HFRS.

## Abbreviations

AKI: Acute kidney injury
ALB: Albumin
ALT: Alanine aminotransferase
APTT: Activated partial thromboplastin time
ARDS: Acute respiratory distress syndrome
AST: Aspartate aminotransferase
AUC: Area under the ROC curve
BUN: Blood urea nitrogen
CI: Confidence interval
EDTA: Ethylenediamine tetraacetic acid
ELISA: Enzyme linked immunosorbent assay
ESL: Endothelial surface layer
Fib: Fibrinogen
GCX: Glycocalyx
GLU: Glucose
HFRS: Hemorrhagic fever with renal syndrome
HTNV: Hantaan virus
MODS: Multiple organ dysfunction syndrome
PGs: Proteoglycans
PLT: Platelets
PT: Prothrombin time
ROC: Receiver operating characteristic
sCD138: Soluble CD138
Scr: Serum creatinine
SIRS: Systemic inflammatory response syndrome
WBC: White blood cells
